# miR-940 is a new biomarker with tumor diagnostic and prognostic value

**DOI:** 10.1016/j.omtn.2021.05.003

**Published:** 2021-05-08

**Authors:** Hongxiang Li, Yin Li, Dongmei Tian, Jiaqian Zhang, Shiwei Duan

**Affiliations:** 1 Medical Genetics Center, School of Medicine, Ningbo University, Ningbo, Zhejiang, China; 2 School of Medicine, Zhejiang University City College, Hangzhou, Zhejiang, China

**Keywords:** miR-940, target gene, pathway, diagnosis, prognosis, drug efficacy

## Abstract

miR-940 is a microRNA located on chromosome 16p13.3, which has varying degrees of expression imbalance in many diseases. It binds to the 3′ untranslated region (UTR) and affects the transcription or post-transcriptional regulation of target protein-coding genes. For a diversity of cellular processes, including cell proliferation, migration, invasion, apoptosis, epithelial-to-mesenchymal transition (EMT), cell cycle, and osteogenic differentiation, miR-940 can affect them not only by regulating protein-coding genes but also long non-coding RNAs (lncRNAs) and circular RNAs (circRNAs) in pathways. Intriguingly, miR-940 participates in four pathways that affect cancer development, including the Wnt/β-catenin pathway, mitogen-activated protein kinase (MAPK) pathway, PD-1 pathway, and phosphatidylinositol 3-kinase (PI3K)-Akt pathway. Importantly, the expression of miR-940 is intimately correlated with the diagnosis and prognosis of tumor patients, as well as to the efficacy of tumor chemotherapy drugs. In conclusion, our main purpose is to outline the expression of miR-940 in various diseases and the molecular biological and cytological functions of target genes in order to reveal its potential diagnostic and prognostic value as well as its predictive value of drug efficacy.

## Graphical abstract

**Figure:**
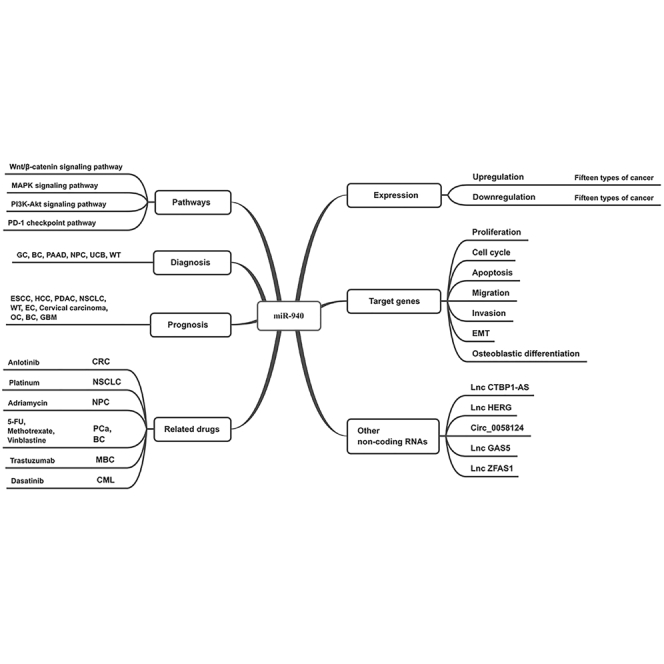

## Teaser

miR-940 is dysregulated in many human cancers, participates in cancer development by regulating target mRNAs, lncRNAs, and circRNAs, and thus affects at least four signaling pathways related to cancer development. miR-940 expression may have a potential diagnostic and prognostic value for cancer patients as well as a predictive value of drug efficacy in cancer treatment.

## Introduction

**Figure 1:**
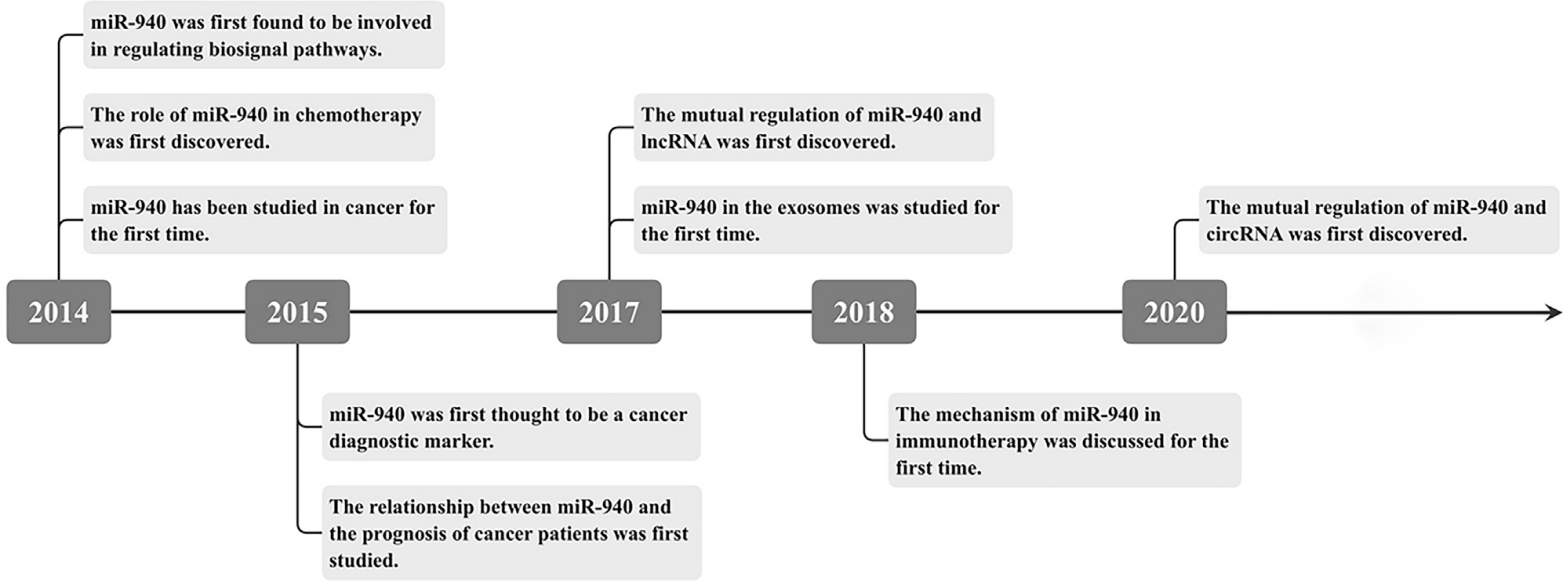
Research history of miR-940 In 2014, miR-940 was studied in cancer for the first time. It was thought that the intervention of miR-940 could be used for sensitive platinum-based chemotherapy, and it was found that miR-940 was involved in regulation of the MAPK signaling pathway. In 2015, a study found that salivary miR-940 has good sensitivity and specificity in the diagnosis of resectable pancreatic cancer. The abnormal expression of miR-940 in PDAC is significantly related to the prognosis of patients. In 2017, it was found that OC cells secreted tumor suppressor miR-940 into the extracellular environment through exosomes. In GBM, miR-940 was found to be a competitive endogenous RNA of lncHERG. In 2018, miR-940 was found to affect the regulation of the PD-1 checkpoint pathway through c-CBL. In 2020, it was found that the miR-940/MAPK1 axis was regulated by circ_0058124 in thyroid carcinoma (TC).

In the genome, only about 1.5% of RNA has the function of protein coding.^1^ Studies in the past decade have found that these remaining non-coding RNAs also have very important biological significance, and they are widely involved in physiological and pathological changes and the occurrence and development of diseases (Figure 1). MicroRNAs (miRNAs) are a class of non-coding RNA with only 20–22 nt. As the most widely studied small molecule in cancer, miRNAs can bind to the 3′ untranslated region (UTR) of target mRNA, resulting in translation barriers or mRNA degradation, thereby controlling cell proliferation, migration, apoptosis, epithelial-to-mesenchymal transition (EMT), and other biological processes. However, miRNAs seem to act as both oncogenes and tumor suppressor genes.^2^

miR-940 is a miRNA located on 16p13.3, and its expression is dysregulated in more than 40 diseases. However, the expression of miR-940 is still controversial in some cancers. On the one hand, downregulation of miR-940 indicates that it plays a role as an oncogene; on the other hand, miR-940 was found to be upregulated, indicating that it also plays a role as a tumor suppressor gene. These diseases include gastric cancer (GC),^3^^,^^4^ hepatocellular carcinoma (HCC),^5^^,^^6^ nasopharyngeal carcinoma (NPC),^7^^,^^8^ glioma,^9^^,^^10^ and other malignant tumors (Table 1).

**Table 1:** Expression and target genes of miR-940 in various cancers

Tumor type	Expression of miR-940	Level	Target gene	Reference
OS	upregulation	tissue and cell	SFRP1	^11^
Bone metastatic lesions	upregulation	cell	ARHGAP1, FAM134A	^12^
ESCC	downregulation	tissue and cell	–	^13^
GC	upregulation	tissue and cell	CBL-b, ZNF24	^3^^,^^14^
	downregulation	tissue and cell	–	^4^
HCC	downregulation	tissue	CXCR2, SPOCK1, ESRRG, H1HR	4, 5, 6^,^^15^, ^16^, ^17^
	downregulation	tissue and cell	–	^6^^,^^16^^,^^17^
CRC	downregulation	tissue and cell	MACC1	^18^
PAAD	upregulation	tissue and cell	GSK3β, SFRP1	^19^^,^^20^
PDAC	downregulation	tissue and cell	MYD88	^21^
OSCC	upregulation	cell	–	^22^
TSCC	downregulation	cell	CXCR2	^23^
NSCLC	downregulation	tissue and cell	c-CBL, FAM83F, SNAI1	24, 25, 26
NPC	upregulation	tissue	–	^7^
	downregulation	tissue	Nestin	^8^
Bladder cancer	upregulation	tissue and cell	INPP4A, GSK-3β	^27^
UCB	upregulation	tissue	–	^28^
PCa	downregulation	tissue and cell	MIEN1	^29^^,^^30^
EC	upregulation	tissue and cell	MRVI1	^31^
TNBC	downregulation	tissue and cell	ZNF24	^32^^,^^33^
MBC	upregulation	tissue	–	^34^
Cervical carcinoma	upregulation	tissue and cell	P27, PTEN	^35^
OC	downregulation	cell	PKC-δ, SRC	^36^^,^^37^
EOC	upregulation	tissue and cell	–	^38^
CML	upregulation	cell	–	^39^
Glioma	upregulation	tissue and cell	KLF9	^10^
	downregulation	tissue	MTHFD2	^9^
GBM	downregulation	tissue and cell	CKS1, ZEB2	40, 41, 42
TC	downregulation	tissue	MAPK1	^43^^,^^44^
Gastric mucosa-associated lymphoid tissue lymphoma	upregulation	tissue	–	^45^

**Figure 2:**
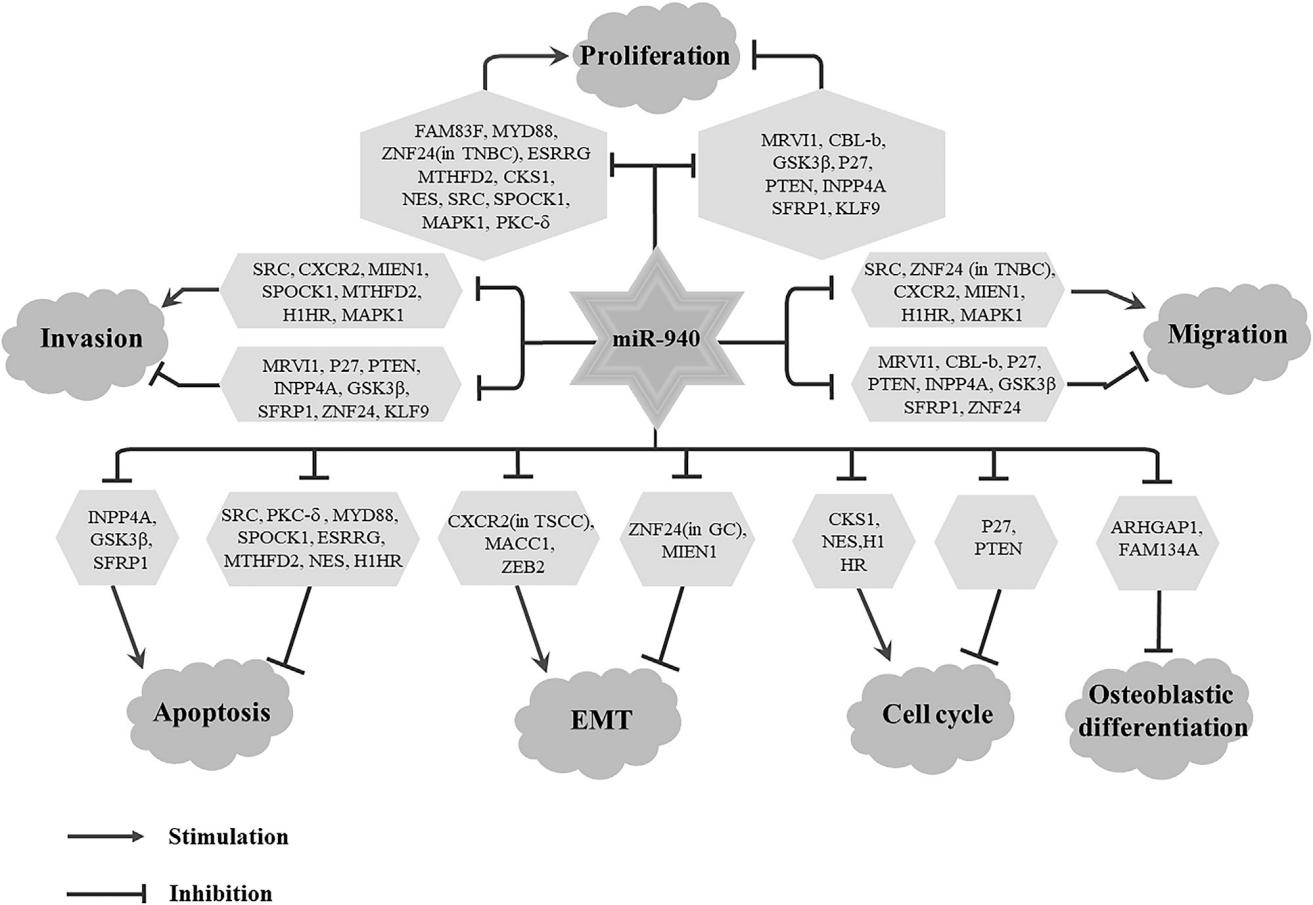
miR-940 affects the biological process of cells through target genes miR-940 affects cell proliferation, migration, invasion, apoptosis, EMT, cell cycle, and osteogenic differentiation by regulating target genes. EMT, epithelial-to-mesenchymal transition. The →-shaped line represents promotion, and the T-shaped line represents inhibition.

OS, osteosarcoma; ESCC, esophageal squamous cell carcinoma; GC, gastric cancer; HCC, hepatocellular carcinoma; CRC, colorectal cancer; PAAD, pancreatic cancer; PDAC, pancreatic ductal adenocarcinoma; OSCC, oral squamous cell carcinoma; TSCC, tongue squamous cell carcinomas; NSCLC, non-small cell lung cancer; NPC, nasopharyngeal carcinoma; UCB, urothelial carcinoma of bladder; PCa, prostatic cancer; EC, endometrial carcinoma; TNBC, triple-negative breast cancer; MBC, HER2^+^ metastatic breast cancer; OC, ovarian cancer; EOC, epithelial ovarian cancer; CML, chronic myeloid leukemia; GBM, glioblastoma; TC, thyroid carcinoma;

Although the mechanism of action of miR-940 is still unclear, current studies have shown that miR-940 not only interregulates with some other non-coding RNAs (Figure 4), but it also downregulates the expression of 29 genes (Table 1). By regulating the functions of many downstream genes, miR-940 can affect cell proliferation,^21^ migration,^23^ invasion,^15^ apoptosis,^11^ cell cycle,^35^ EMT,^40^ osteogenic differentiation,^12^ and other biological processes (Figure 2).

**Figure 3:**
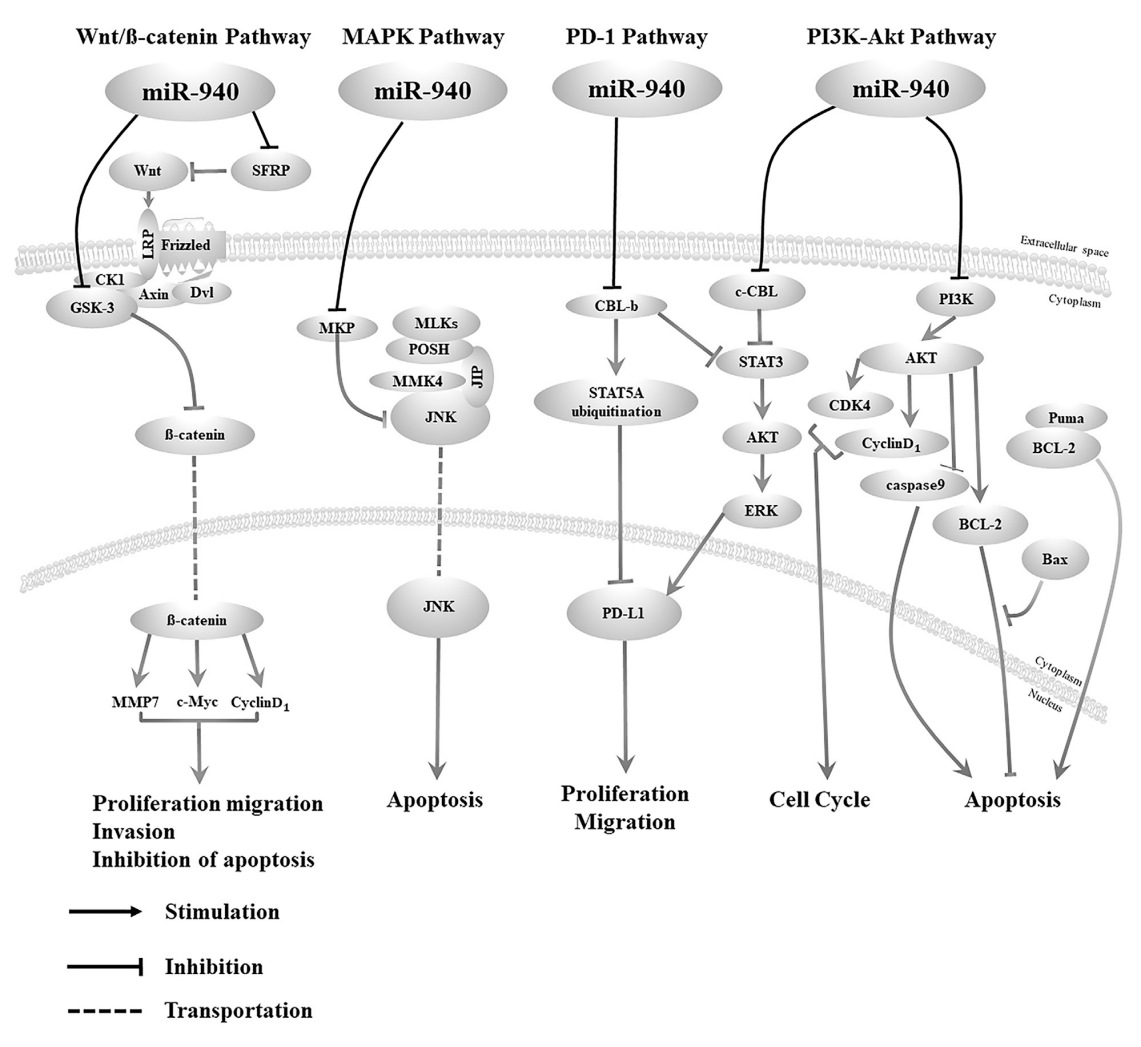
miR-940 regulates the biological processes of cells by affecting signaling pathways miR-940 regulates the signal molecules in the Wnt/β-catenin signaling pathway, MAPK signaling pathway, PD-1 checkpoint pathway, and PI3K-Akt signaling pathway.

miR-940 can indirectly affect the functions of multiple signaling pathways by regulating target genes. These target genes involve the Wnt/β-catenin pathway,^11^^,^^19^^,^^27^ mitogen-activated protein kinase (MAPK) pathway,^46^ PD-1 pathway,^3^ and phosphatidylinositol 3-kinase (PI3K)-Akt pathway (Figure 3).^24^^,^^47^

miR-940 also has diagnostic and prognostic value in different diseases of the respiratory system, digestive system, urinary system, reproductive system, nervous system, and endocrine system (Tables 3, 4, and 5). The expression of miR-940 is also related to drug sensitivity^8^ and drug resistance,^12^ which has potential clinical value in predicting the therapeutic effect of drugs.^34^ These findings emphasize that miR-940 plays an important role from the occurrence and development of cancer to the diagnosis and treatment of cancer, and it is of practical significance to explore the mechanism of miR-940 in this process.

**Table 3:** Diagnostic value of miR-940 in different diseases

Types	Ways	Samples	Results	Reference
GC	comparison of gastric cancer patients and non-gastric cancer patients	plasma from 115 patients with gastric cancer and 105 healthy controls	sensitivity = 81.25%, specificity = 98.57%, AUC = 0.9657 (95% CI = 0.9400–0.9915)	^4^
BC and TNBC	comparison of breast cancer patients and non-breast cancer patients, and miR-940 high-expression patients and low-expression patients	128 breast cancer patients	sensitivity = 94.5%, specificity of 78.6%, AUC = 0.905	^33^
PAAD	miR-3679-5p and miR-940; comparison of pancreatic cancer patients and non-pancreatic cancer patients (benign pancreatic tumor and healthy people)	saliva from 40 pancreatic cancer patients and 60 non-cancer patients (20 benign pancreatic cancer and 40 healthy controls)	sensitivity = 70.0%, specificity = 70.0%, AUC = 0.763	^20^
Submucosal invasive gastric cancer	four-serum miRNA signature including miR-153-3p, miR-708, miR-940, and miR-375	114 biopsy specimens	AUROC = 0.792 (95% CI = 0.731–0.873)	^48^
NPC	three-serum miRNA signature including miR-548q and miR-940	55 NPC patients and 45 non-NPC patients	miR-548q + miR-940, sensitivity = 94.0%, specificity = 92.5%, AUC = 0.972 (95% CI, 0.913–0.995)	^7^
UCB	four-miRNA signature including miR-26a, miR-93, miR-191, and miR-940	urine samples from 85 patients with UCB and 45 controls	At 95% CI (56%–84%), sensitivity = 70%; at 95% CI (74%–95%), specificity = 84%, AUC = 85.8%	^28^
WT	five-serum miRNA signature including hsa-miR-149, hsa-miR-7112, hsa-miR-940, hsa-miR-1248, and hsa-miR-490, high-expression patients and low-expression patients	127 patients with WT	AUC = 0.767; it has good specificity and sensitivity	^49^

BC, breast cancer; AUC, area under the curve; AUROC, area under the receiver operating characteristic curve; CI, confidence interval; PAAD, pancreatic cancer; WT, Wilms tumor.

**Table 4:** Prognostic value of miR-940 in different diseases

Types	Samples	Prognosis of miR-940 overexpression	Reference
ESCC	210 ESCC patients	good	^13^
HCC	362 HCC patients	poor	^5^
	46 HCC patients	good	^6^
	377 HCC patients	good	^17^
	23 HCC patients	good	^16^
PDAC	78 PDAC patients	good	^21^
NSCLC	72 NSCLC patients	good	^25^
WT	132 WT patients	poor	^49^
EC	546 EC patients	poor	^31^
Cervical carcinoma	83 cervical carcinoma patients	poor	^35^
OC	471 OC patients	good	^37^
BC	128 BC patients	good	^33^
GBM	198 GBM patients	poor	^41^

**Table 5:** The relationship between miR-940 and clinical pathology

Types	Samples	Clinicopathological parameters	Reference
ESCC	210 ESCC patients	tumor differentiation, lymph node metastasis, TNM stage	^13^
GC	115 patients with GC and 105 healthy controls	TNM stage	^4^
HCC	46 HCC patients	Edmondson grade, tumor microsatellite, multiple tumors, vascular invasion	^6^
NSCLC	72 NSCLC patients	tumor stage, tumor size	^25^
EC	546 EC patients	age, grade, death	^31^
Cervical carcinoma	83 Cervical carcinoma patients	cervical cancer progression	^35^
BC and TNBC	128 BC patients	lymph node metastasis, TNM stage	^33^
	38 TNBC patients		
PTC	266 PTC patients	bilateral tumor, multicentricity, extrathyroidal invasion, cervical lymph node metastasis, distant metastasis, clinical advanced stages (III/IV)	^44^

## miR-940 is an important molecule in human cancers

### miR-940 is abnormally expressed in different cancers

The abnormal expression of miR-940 in different diseases is inconsistent. Abnormal expression of miR-940 has been found in 26 cancers (Table 1).

The evidence so far suggests that miR-940 in osteosarcoma (OS), bone metastatic lesions, GC, HCC, pancreatic adenocarcinoma (PAAD), oral squamous cell carcinoma (OSCC), NPC, bladder cancer, urothelial carcinoma of bladder (UCB), endometrial carcinoma (EC), HER2^+^ metastatic breast cancer (MBC), cervical carcinoma, epithelial ovarian cancer (EOC), chronic myeloid leukemia (CML), glioma, and mucosa-associated lymphoid tissue lymphoma is highly expressed in tissues or cell lines. At the same time, miR-940 was also found to be poorly expressed in the tissues and cell lines of esophageal squamous cell carcinoma (ESCC), GC, HCC, colorectal cancer (CRC), pancreatic ductal adenocarcinoma (PDAC), tongue squamous cell carcinomas (TSCC), non-small cell lung cancer (NSCLC), NPC, prostate cancer (PCa), triple-negative breast cancer (TNBC), ovarian cancer (OC), glioma, glioblastoma (GBM), and thyroid carcinoma (TC). The above results were verified by PCR.

The expression profile of miR-940 in cancer is so extensive that it can be seen that the expression of miR-940 in GC, HCC, NPC, and glioma presents contradictory results. In Table 2, we summarized the information on the samples or cell lines with differential expression of miR-940, and how the differences in the number or quality of samples and cell lines may affect the expression of miR-940. First, the number of samples collected in each study is small, so it was necessary to expand the sample for verification. Second, differences in cell lines adopted may affect the experimental results. Studies have shown that the expression of miR-940 varies greatly in GC cell lines. MGC803 and HGC27 cells express a low level of miR-940, while SNU16, MKN87, KATO III, and SGC7901 cells express a high level of miR-940,^14^ and the same situation has been confirmed in HCC and glioma.^6^^,^^10^^,^^16^ In addition, the expression of miR-940 may also be related to tissue heterogeneity, and the expression level of miR-940 in HCC tissues with vascular invasion was higher than that in HCC tissues with non-vascular invasion.^5^

**Table 2:** Comparison of study samples in diseases with inconsistent miR-940 expression

Types	Sample type	Name and quantity	The expression of miR-940		Reference
GC	tissues	38 pairs of GC tissues and paracancerous tissues	higher in GC		^14^
	cell line	the normal gastric membrane cell line GES-1 and GC cell lines MGC803, HGC27, SNU16, MKN87, KATO III, and SGC7901	higher in SNU16, MKN87, KATO III, and SGC7901; lower in GES-1, MGC803, and HGC27		^14^
	plasma	plasma from 30 healthy people and 30 patients with gastric cancer	lower in plasma of GC		^4^
	tissues	34 pairs of GC tissues and paracancerous tissues	lower in GC		^4^
HCC	tissues	81 patients with and 91 without vascular invasion hepatocellular carcinoma	higher in tissues with vascular invasion; lower in tissues without vascular invasion		^5^
	tissues	46 pairs of HCC tissues and paracancerous tissues	lower in HCC		^6^
	cell line	normal cells (HL-7702) and HCC cells (MHCC-97H, SMMC-7721(	higher in HL-7702; lower in MHCC-97H and SMMC-7721		^6^
	tissues	46 pairs of HCC tissues and paracancerous tissues	lower in HCC		^17^
	cell line	normal cells (HL-7702) and HCC cells (HepG2, Hep3b)	higher in HL-7702; lower in HepG2 and Hep3b		^17^
	tissues	23 pairs of HCC tissues and paracancerous tissues	lower in HCC		^16^
	cell line	normal cells (Chang, L-02) and HCC cells (SMMC-7721, Huh-7, HepG2, Hep3B)	higher in Chang, L-02, HepG2, and Hep3B; lower in SMMC-7721 and Huh-7		^16^
NPC	tissues	55 NPC patients and 45 non-cancerous controls	higher in NPC		^7^
	tissues	28 pairs of NPC tissues and para-cancerous tissues	lower in NPC		^8^
Glioma	tissues	23 pairs of glioma tissues and paracancerous tissues (14 males, 9 females; age range, 47–72 years)	higher in glioma		^10^
	cell line	normal human astrocytes NHA and glioma cells (U251, U87, T98G, and LN229)	higher in U251, U87, T98G, and LN229; lower in NHA		^10^
	tissues	5 normal tissues and 525 glioma tissues	lower in glioma		^9^
	tissues	5 normal tissues and 34 glioma tissues	lower in glioma.		^9^

### The regulation of miR-940 on target genes in oncogenesis

A total of 29 miR-940 target genes can be found in skeletal diseases and diseases of the digestive system, respiratory system, urinary system, reproductive system, cardiovascular system, nervous system, and endocrine system. All of the target genes are negatively regulated by miR-940, but their effects on cancer cells are negatively regulated (Figure 2).

#### Role of miR-940 in cell proliferation and cell cycle

It is clear that the presence of a large number of miR-940 target genes are associated with cell proliferation and cell cycle. miR-940 was expressed at a low level in NSCLC cell lines (H1299 and SK-MS-1), while miR-940 inhibited the expression of FAM83F.^25^ FAM83F activates the Wnt signaling pathway, which is known to increase tumor proliferation.^50^ miR-940 mimics reduced cell proliferation by inhibiting FAM83F. Overexpressed miR-940 also downregulates proto-oncogene tyrosine protein kinase (SRC) and its downstream proteins such as FAK, paxillin, and Akt, thereby inhibiting the proliferation of ovarian cancer cell lines (SKOV3-IP1, HeyA8, HeyA8-MDR).^50^ In other ovarian cancer cell lines (OVCAR3), downregulation of miR-940 and upregulation of its target gene, protein kinase C (PKC)-δ, were found.^36^ PKC-δ is an isoform of PKC and is involved in signal transduction. Inhibition of PKC-δ has been reported to block the G_0_/G_1_ transformation of the cell cycle to reduce cell proliferation, while downregulation of miR-940 suggests that PKC-δ is involved in promoting ovarian cancer cell proliferation.^51^^,^^52^ These results suggest that miR-940 plays an inhibitory role in ovarian cancer by inhibiting cell proliferation. In some other cancers, such as TNBC and PDAC, downregulation of miR-940 and upregulation of its target genes have also been found. As an endogenous zinc finger transcription factor, ZNF24, directly binds the CTNNB1 promoter and activates β-catenin to promote cell proliferation.^53^ MyD88 is an essential adaptor of interleukin (IL)-1 and Toll-like receptor (TLR) signal transduction, which plays an important role in cytokine response and is related to cell proliferation.^54^ miR-940 prevented overproliferation of TNBC cell lines (MDA-MB-231 and BT-549) and PDAC cell lines (PANC1 and SW1990) by inhibiting these two genes.^54^ In hepatocellular cancer cell lines (HepG2 and Hep3b), miR-940 is also downregulated,^17^ and the highly expressed SPOCK1 is a possible cause of promoting the proliferation of HCC cells. SPOCK1 has been reported to increase the proliferation of NSCLC cells through the Wnt/β-catenin signaling pathway.^55^ In other hepatocellular cancer cell lines (SMMC-7721 and Huh-7), overexpression of miR-940 targeted ESRRG to inhibit cell proliferation.^16^ miR-940 also inhibits H1HR in HCC. H1HR, as a member of the rhodopsin like G protein-coupled receptor family, can activate the protein kinase A (PKA) pathway. By inhibiting H1HR, miR-940 blocked the cell cycle in the G_1_ phase and inhibited the G_1_-S phase transition. In nude mice, H1HR overexpression also increased tumorigenicity.^15^ Overexpression experiments of miR-940 showed that it could reduce the number of colonies in thyroid cancer cell lines (TPC-1 and HTH83),^43^ which might be related to its inhibition of the proliferation-related gene MAPK1. MAPK1 is a well-known cell proliferation regulator that is essential for cell proliferation and is involved in the transduction of the MAPK signaling pathway.^56^ In glioma cell lines (U87 and LN229), miR-940 negatively regulated MTHFD2^9^ and CKS1^41^ to inhibit tumor cell proliferation. Among them, MTHFD2 affects cell proliferation through the pathway of folate metabolism, and deficiency of MTHFD2 leads to impairment of one-carbon unit utilization, which is an important reason for cell proliferation inhibition.^57^ CKS1 is dysregulated in a variety of cancers and regulates cell cycle and proliferation by interacting with cyclin-dependent kinases (CDKs) and is involved in cell G_1_/S transformation.^58^ miR-940 mimics reduced the expression of CKS1 and blocked cells in the G_0_/G_1_ phase, inhibiting cell proliferation.^41^ In NPC, the target gene Nestin is also related to the cell cycle. Nestin depletion leads to the activation of cyclin-dependent kinase 5 (CDK5) and accelerates tumor senescence.^59^ The low expression of miR-940 in a NPC cell line (5-8F) is the reason why Nestin promotes cell proliferation.^8^

However, in some cancers, miR-940 acts as an oncogene to promote cell proliferation. miR-940 was significantly overexpressed in an endometrial cancer cell line (RL95-2) and tissues. MRVI1 is a tumor suppressor gene that is transcriptionally activated by the tumor suppressor p53 and has been reported to inhibit the proliferation of CRC cells.^60^ Overexpressed miR-940 inhibited MRVI1 and promoted the proliferation of endometrial cancer cells.^31^ miR-940 also inhibits another tumor suppressor gene named CBL-b, thereby promoting the proliferation of GC cell lines (MGC803 and AGS).^3^ Overexpression of miR-940 may promote the proliferation of bladder cancer cells by targeting INPP4A and GSK3β, and the inhibitory effect of miR-940 on the proliferation of bladder cancer cells may be mediated by the activated Wnt/β-catenin signaling pathway.^27^ In an osteosarcoma cell line (U2OS), miR-940’s targeted inhibition of SFRP1 to promote cell proliferation is also mediated by activation of the Wnt/β-catenin signaling pathway.^11^ In addition, miR-940 has also been reported to be highly expressed in glioma cell lines (U251 and U87) and to promote cell proliferation by targeting down KLF9 expression.^10^ Two tumor suppressor genes, p27 and PTEN, which are often dysregulated in hematological malignancies, have also been reported in cervical cancer cell lines (SiHa and HeLa). PTEN acts as PIP3’s phosphatase to negatively regulate the PI3K/Akt pathway, while p27 is a cyclin-dependent kinase inhibitor that regulates G_1_-to-S transitions by binding to cyclin-dependent kinases and regulating their activity.^61^ miR-940 inhibits their expression at the post-transcriptional level and reduces the level of cell protein cycle D1, accelerates the cell cycle, and promotes cell proliferation.^35^

#### Role of miR-940 in cell apoptosis

Apoptosis (the programmed cell death 1 [PD-1] form) not only affects the number of tumors, but it also is of great significance to the transfer of tumor cells, and malignant tumor cells must overcome various forms of cell death to be transferred.^62^ miRNAs are important factors in the regulation of cell apoptosis, involving numerous signaling pathways and genes. Current studies show that miR-940 has a certain effect on tumor cell apoptosis.^63^ Wang et al.^27^ found that miR-940 as an oncogene can target INPP4A or GSK3β and inhibit T24 apoptosis in bladder cancer cells by activating the Wnt/β-catenin pathway. In osteosarcoma cells U2OS, miR-940 also inhibited apoptosis by inhibiting SFRP1 and activating the Wnt/β-catenin signaling pathway.^11^ miR-940 also acts as a tumor suppressor gene to increase cell apoptosis. It has been reported that the tumor suppressor miR-940 is secreted by malignant ovarian cancer cells (HEYA8) into the extracellular fluid via the exosome pathway to maintain its tumorigenic phenotype, and miR-940 can increase the caspase-dependent apoptosis pathway by inhibiting SRC.^37^ In another ovarian cancer cell line, OVCAR3, miR-940 induced apoptosis by targeting PKC-δ.^36^ In pancreatic duct adenocarcinoma, miR-940 is underexpressed, and cell assays and dual-luciferase assays confirmed that miR-940 targets MyD88 and induces apoptosis in pancreatic duct adenocarcinoma cell lines (PANC1 and SW1990).^21^ Similarly, in hepatocellular cancer cell lines (HepG2 and Hep3B), miR-940 promotes apoptosis by targeting SPOK1.^17^ miR-940 was also underexpressed in other hepatocellular cancer cell lines (SMMC-7721, Huh-7, SNU-368, and HLE), and simulated transfection experiments showed that miR-940 could inhibit estrogen-associated receptor γ (ESRG) and H1HR to induce apoptosis.^15^^,^^16^ MTHFD2 is a key enzyme in folate metabolism and is involved in cancer proliferation, migration, and maintenance of redox homeostasis.^64^^,^^65^ In the miRNA simulation experiment of glioma cell lines (U87 and U118), the inhibition of miR-940 on MTHFD2 resulted in abnormal intracellular carbon metabolism and promoted apoptosis of glioma cells.^9^ In addition, miR-940 also increased the phosphorylation of DNA damage-reactive proteins by downregulating Nestin in nasopharyngeal cancer cell lines (5-8F), which resulted in the accumulation of DNA damage, delayed DNA repair, and promoted apoptosis.^8^

#### Role of miR-940 in cancer migration and invasion

The metastatic and invasive nature of malignant tumors is defined as the process of the metastasis of independent cells from the primary tumor to the distal organs via blood vessels or lymphatics, which is an important cause of cancer death.^66^ miR-940 inhibits tumor migration and invasion by regulating target genes. First, as a tumor suppressor, miR-940 can target the proto-oncogene SRC kinase and its downstream genes, thereby inhibiting the migration and invasion of ovarian cancer cell lines (HeyA8, HeyA8-MDR, and SKOV3IP1).^37^ Src is not only involved in proliferation and apoptosis of tumor cells, but it is also involved in cell migration by regulating the phosphorylation of EMT-associated intermediate filaments (vimentin intermediate filaments [VIFs]).^67^ miR-940 regulates HCC migration and proliferation by inhibiting CXCR2 (cell lines MHCC97H and SMMC-7721), H1HR (cell lines SNU-368 and HLE), and SPOCK1 (cell lines HepG2 and Hep3B).^6^^,^^15^^,^^17^^,^^67^ As a chemokine, CXCR2 has been reported to promote the migration, invasion, and EMT of thyroid papillary carcinoma cells through the β-catenin pathway.^68^ Low expression of miR-940 and high expression of CXCR2 in HCC may contribute to the high metastasis rate of HCC.^6^ H1HR is a histamine receptor, and the application of histamine receptor blockers can reduce the expression of von Willebrand factor (vWF), vascular endothelial growth factor (VEGF)A, and EMT-related proteins, suggesting that H1HR may be an important mediator of cell metastasis and invasion.^69^ SPOCK1 has been reported as a gene associated with migration and invasion of a variety of cancers, affecting EMT through the PI3K/Akt and Wnt/β-catenin pathways, and enhancing cell migration and invasion characteristics.^55^^,^^70^ miR-940 inhibits the migration and invasion of prostate cancer cell lines (DU-145 and PC-3) by targeting MIEN1.^29^ Similar results were found in thyroid cancer cell lines (TPC-1 and HTH83), with low expression of miR-940 and high expression of target gene MAPK1. MAPK1 activates the extracellular signal-regulated kinase (ERK)1/2 signaling pathway and promotes thyroid migration and invasion.^43^ MTHFD2 is involved in folate metabolism and is known to modulate vimentin expression to promote migration and invasion of renal cell carcinoma.^71^ miR-940 simulators inhibit the expression of MTHFD2 and thus inhibit the invasion ability of glioma cell lines (U87 and U118).^9^ ZNF24 is a transcription factor closely related to angiogenesis, and inhibition of ZNF24 has been reported to significantly reduce the expression levels of VEGFR2 and MMP-2 in breast cancer (BC) cells.^72^ miR-940 was expressed at a low level in TNBC cell lines (MDA-MB-231 and BT-549), and its target gene ZNF24 was highly expressed and promoted cell migration.^32^

However, miR-940 can also play a role as an oncogene in promoting tumor migration and invasion. The feedback loop formed by MRVI1 and ATF3 activates the tumor suppressor RASSF1 and further activates the Hippo pathway. The Hippo pathway is known to be a complex signaling network that regulates cell proliferation, differentiation, and migration of developmental organs.^73^^,^^74^ In an endometrial cancer cell line (RL95-2), miR-940 is highly expressed, which reduces the inhibitory activity of MRVI1 on migration and invasion by downregulating MRVI1, and ultimately promotes tumor metastasis.^31^ p27 and PTEN are two tumor suppressor genes that are closely linked through the Akt signaling pathway and have synergistic effects in tumor suppression.^61^ In cervical cancer cell lines (Siha and HeLa), miR-940 also showed a trend of overexpression and downregulated p27 and PTEN, thereby promoting cell migration and invasion.^35^ The classical Wnt/β-catenin signaling pathway is a complex and conserved signaling pathway, which plays an important role in tumor growth and metastasis. Its signaling is also regulated by many factors and is often abnormally activated in malignant diseases.^75^ In a bladder cancer cell line (T24), miR-940 was overexpressed and inhibited INPP4A and GSK3β, which led to abnormal activation of the Wnt/β-catenin pathway to promote cell migration and invasion.^27^ In the human osteosarcoma cell line (U2OS), the Wnt/β-catenin signaling pathway was also abnormally activated by highly expressed miR-940 due to SFRP1 inhibition. Previous reports have shown that SFRP1 inhibits the Wnt signaling pathway in at least four ways, including direct binding of the NTR domain or indirect blocking of the CDR motifs. miR-940 inhibits SFRP1 activation of the Wnt pathway and promotes migration and invasion.^11^ Interestingly, contrary to the previous results of ZNF24 promoting cancer proliferation, ZNF24 showed a tumor suppressive effect in GC cell lines (MGC803 and HGC27). ZNF24 has been reported to reduce angiogenesis by directly binding to the VEGF proximal promoter. Overexpressed miR-940 promotes the migration and invasion of GC by downregulating the expression of ZNF24.^14^ In GC cell lines (MGC803, AGS, NCI-N87, and MKN74), miR-940 also regulates the PD-1 checkpoint pathway through the CBL-b/Stat5a axis, thereby promoting cell migration.^3^ KLF9 has previously been identified as an important molecule that reduces the invasion of several cancers by inhibiting the activity of the MMP family promoter.^76^^,^^77^ Overexpression of miR-940 in glioma cells (U251 and U87) and downregulation of KLF9 promoted tumor invasion.^10^

#### Role of miR-940 in EMT

EMT is a biological process in which tumor cells transform into cells with stromal phenotypes, which significantly promotes tumor migration and invasion. It is characterized by reduced expression of cell adhesion molecules (such as E-cadherin) and keratin, and increased expression of vimentin and N-cadherin.^78^^,^^79^ Ma et al.^23^ found that miR-940 attenuates nuclear factor κB (NF-κB) and IL-8-induced EMT by inhibiting CXCR2 in tongue squamous cell cancer cell lines (TSCCA and TCA8113). MACC1 is a gene associated with metastasis of colon cancer and is associated with malignant phenotypes of colon cancer.^80^ miR-940 inhibits the expression of EMT-related proteins in CRC cells (SW620 and Lovo cells) by targeting MACC1, and it reduces the migration and invasion of cancer cells.^18^ ZEB2 is another gene closely related to EMT, which can promote the EMT of melanoma, breast cancer, and other tumors.^81^^,^^82^ In glioma cell lines (U87 and LN229), miR-940 was expressed at a low level, while ZEB2 was expressed at a high level, suggesting that miR-940 negatively regulates the EMT process of glioma.^40^ However, Liu et al.^14^ found that miR-940 promoted EMT of GC cell lines (HGC27 and MGC803) by targeting ZNF24. ZNF24 is a transcriptional regulator that reduces angiogenesis by inhibiting VEGFA and has been reported to increase Twist1-induced EMT in prostate cancer.^83^ In addition, miR-940 also induces EMT in DU-145 cells by increasing the expression of E-cadherin by targeting MIEN1 in prostate cancer.^29^

#### Role of miR-940 in osteoblastic differentiation

miRNAs affect the tumor microenvironment and alter cancer phenotypes through the exosome pathway.^84^ Bone metastatic cells from prostate cancer can secrete miR-940 to affect mesenchymal stem cells and promote their osteogenic differentiation, which is due to the inhibitory effect of miR-940 on ARHGAP1 and FAM134A and the increased osteogenic differentiation of stem cells by promoting the RhoA/ROCK pathway.^12^

## miR-940-related signal pathways

### The role of miR-940 in regulating the Wnt/β-catenin signaling pathway

Abnormalities in the Wnt/β-catenin signaling pathway promote the renewal, proliferation, and differentiation of cancer stem cells and play an important role in tumor genesis and development.^85^ miR-940 is significantly upregulated in bladder cancer tissues and cells, and its overexpression can significantly increase the protein expression levels of c-Myc, cyclin D1, and β-catenin. The effect of miR-940 can be reversed by using the Wnt/β-catenin signaling pathway inhibitor XAV939.^27^ A study has also pointed out that in osteosarcoma tissue, the upregulation of miR-940 negatively regulates the expression of SFRP1, while the expression of β-catenin and cyclin D1 increases, which regulates participation in the Wnt/β-catenin signaling pathway.^11^ Similarly, Yang et al.^19^ also demonstrated that miR-940 targeted negative regulation of GSK3β and SFRP1 genes in PADD, leading to the activation of the Wnt/β-catenin pathway. In summary, in a variety of cancers including bladder cancer, osteosarcoma, and PAAD, miR-940 has been shown to activate the Wnt/β-catenin signaling pathway, thereby promoting cancer cell proliferation, apoptosis, and invasion, and inhibiting apoptosis (Figure 3).

### The role of miR-940 in regulating the MAPK signaling pathway

The MAPK cascade is highly conserved and involves a variety of cellular biological processes, including proliferation, differentiation, and migration. The expression of miR-940 was significantly increased in RIP1 knockdown cells and promoted the apoptosis of NSCLC cells through the MAPK pathway, thereby increasing the sensitivity of cancer cells to cisplatin. Inhibiting the activity of miR-940 can enhance the expression of MKP1, thereby reducing the activity of JNK induced by cisplatin (Figure 3).^46^

### The role of miR-940 in regulating the PD-1 checkpoint pathway

PD-1 and its ligand (PD-L1) are regulatory physiological immune checkpoints. The combination of PD-L1 on cancer cells and PD-1 on immune cells is conducive to the immune escape of cancer. Therefore, blocking PD-1/PD-L1 is conducive to the clearance of cancer cells.^86^ PD-L1 is a ligand for programmed death, which not only inhibits tumors, but also promotes the proliferation and migration of cancer cells.^87^ miR-940 can target CBL-b in GC, thereby promoting the ubiquitination of STAT5A and inhibiting PD-L1.^3^ In NSCLC, miR-940 upregulates PD-L1 by inhibiting c-CBL to promote STAT3/AKT/ERK signal transduction (Figure 3).^24^

### The role of miR-940 in regulating PI3K-Akt signaling pathway

The PI3K-Akt signaling pathway is stimulated by many types of cellular or toxic agents. Dysfunction of this pathway can lead to changes in basic cellular function.^88^ In NSCLC, miR-940 targets c-CBL, and c-CBL can inhibit STAT3, thereby inactivating the AKT/ERK pathway, and indirectly inhibiting PD-L1.^24^ In cervical cancer (cervical squamous cell carcinoma and endocervical adenocarcinoma [CESC]), miR-940 reduces the expression levels of cyclin D1 and CDK4 by inhibiting the PI3K/Akt pathway.^47^ miR-940 can also promote apoptosis-related factor caspase-9, inhibiting BCL-2 through the PI3K/Akt pathway, and achieve the effect of promoting cancer cell apoptosis.^47^ In addition, miR-940 can also significantly increase the expression levels of Puma and Bax.^47^ Bax may form a heterodimer with Bcl-2, preventing Bcl-2 from inhibiting apoptosis.^89^ Puma binds to Bcl-2 to induce the release of cytochrome *c* and activate cell apoptosis (Figure 3).^90^

### Interaction of miR-940 with other non-coding RNAs

**Figure 4:**
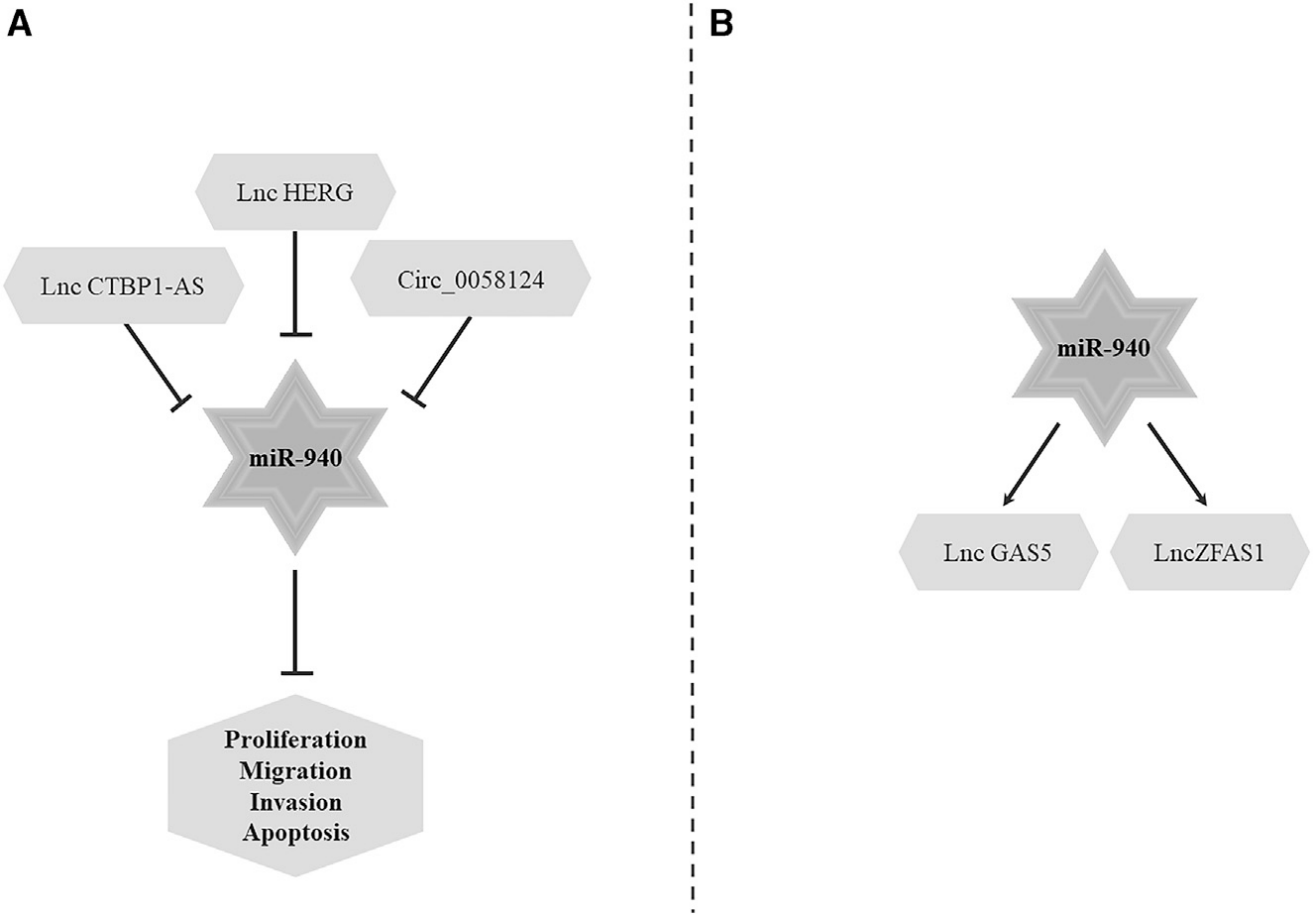
The regulatory role between miR-940 and other non-coding RNAs The interaction of miR-940 with other non-coding RNAs can affect cell proliferation, migration, invasion, and apoptosis. The →-shaped line represents promotion, and the T-shaped line represents inhibition.

In prostate cancer, the expression of miR-940, lncGAS5, and lncZFAS1 is upregulated, and miR-940 promotes the expression of lncGAS5 and lncZFAS1 by targeting NAA10 and RPL28 genes.^91^ In addition, miRNA can also be targeted and regulated by long non-coding RNA (lncRNA). In GBM, lncHERG can target miR-940 to inhibit cell proliferation, migration, invasion, and apoptosis.^42^ In breast cancer, miR-940 is the downstream target of lncRNA CTBP1-AS. Overexpressed lncRNA CTBP1-AS can inhibit cell apoptosis by sponging miR-940, thereby promoting the proliferation, invasion, and migration of breast cancer cells.^92^ In addition, in thyroid cancer, miR-940 can bind to circ_0058124. Overexpression of miR-940 inhibits the growth of thyroid cancer cell lines, while overexpression of circ_0058124 can restore the growth of thyroid carcinoma cell lines by regulating the circ_0058124/miR-940/MAPK1 axis (Figure 4).^43^

## miR-940 as a potential marker and therapeutic target

In many studies, miR-940 has been found to be closely related to the occurrence and development of certain diseases, and it has good diagnostic and prognostic value. The expression level of miR-940 is also closely related to the clinicopathological conditions of patients. miR-940 is expected to be a diagnostic and prognostic biomarker for certain diseases, including NSCLC, NPC, and endometrial cancer, among others.

### The diagnostic and prognostic value of miR-940 in different diseases

Studies have shown that miR-940 has high sensitivity and specificity in the diagnosis of diseases such as GC^4^ and breast cancer.^33^ In addition, miR-940 can also be used as a signature model with other miRNAs for the diagnosis of submucosal invasive GC,^48^ PAAD,^20^ NPC,^7^ UCB,^28^ Wilms tumor (WT),^49^ and other diseases (Table 3).

In addition, in ESCC,^13^ HCC,^6^^,^^16^^,^^17^ PDAC,^21^ NSCLC,^25^ and ovarian cancer,^37^ the high expression of miR-940 is significantly related to the better prognosis of patients. On the contrary, some studies have found that in other tumors, including HCC,^5^ colon adenocarcinoma (COAD),^93^ WT,^49^ endometrial carcinoma,^31^ cervical carcinoma,^35^ BC,^33^ and GBM,^41^ the prognosis of patients with high expression of miR-940 is significantly worse than that of patients with low expression of miR-940. In addition, miR-940 can also be combined with other miRNAs to predict the prognosis of patients. In COAD, a risk assessment model composed of 10 miRNAs including miR-940 is capable of assessing the risk of colon adenocarcinoma patients, and patients with high-risk scores are significantly associated with poor prognosis.^93^ Five differentially expressed miRNAs, including miR-940, can be used for prognostic evaluation of WT patients (Table 4).^49^

### The relationship between miR-940 and clinical pathology

In ESCC patients with lymph node metastasis, TNM (tumor, lymph node, metastasis) stage and miR-940 expression are independent risk factors that affect the prognosis of patients. The low expression of miR-940 is closely related to poor tumor differentiation, positive lymph node metastasis, and clinical stage.^13^ In addition, the expression of miR-940 in the plasma of patients with GC of various stages is significantly different from that of the control group, and the expression level of plasma miR-940 in patients with stage IV GC is higher than that of patients with stages I, II, and III GC.^4^ In HCC, low miR-940 is significantly associated with higher Edmondson grade, microsatellite tumors or multiple tumors, and advanced vascular invasion, as well as other clinical indicators.^6^ In patients with NSCLC, the expression of miR-940 is negatively correlated with the tumor stage and tumor size.^25^ In endometrial cancer samples, the expression level of miR-940 was significantly correlated with age, grade, and overall survival.^31^ In patients with cervical cancer, the expression of miR-940 is negatively correlated with the tumor stage.^35^ Among breast cancer patients, miR-940 and TNM stage are closely related to the overall survival of breast cancer patients. Among patients with TNBC, the overall survival of patients with high expression of miR-940 is still higher.^33^ In patients with papillary thyroid carcinoma (PTC), the low expression level of miR-940 is associated with tumor distribution, tumor metastasis, and advanced tumor TNM stage (Table 5).^44^

### The effect of miR-940 on drug treatment

In CRC, miR-940 enhances the sensitivity of CRC cells to anlotinib by targeting MACC1.^18^ In NSCLC, the miR-940/MKP1/JNK axis can increase the sensitivity of platinum-based chemotherapy.^46^ In addition, miR-940 can target to inhibit Nestin, cause spontaneous accumulation of DNA damage, and increase the sensitivity of NPC to radiation and adriamycin.^8^ However, miR-940 can enhance the resistance of cancer cells to chemotherapy drugs such as 5-FU, methotrexate, and vinblastine, leading to the invasive bone metastases of prostate cancer and breast cancer.^12^ The expression level of miR-940 can also be used to predict the therapeutic effect of drugs. A four-miRNA panel (including miR-940, miR-451a, miR-16-5p, and miR-17-3p) has been shown to predict trastuzumab response in HER2^+^ metastatic breast cancer patients.^34^ In chronic myeloid leukemia, after dasatinib treatment, the expression of miR-940 is significantly reduced, and the expression change of miR-940 can be used as a potential therapeutic target for chronic myeloid leukemia patients.^39^

## Summary of research methods

The miRNA microarray is applied to read the miRNA expression profile to screen out the differentially expressed miRNAs. Its advantage lies in its huge throughput, but it has a relative lack of sensitivity and specificity.^94^ With the advent of the era of big data, public databases have become important data sources for bioinformatics research. In the literature collected by us, The Cancer Genome Atlas (TCGA) (https://www.cancer.gov/tcga/), GEO (https://www.ncbi.nlm.nih.gov/geo/), Chinese Glioma Genome Atlas (CGGA) (http://www.cgga.org.cn/), and TARGET databases are commonly used to verify experimental results. In addition, authors also applied a miRNA data integration portal mirDIP (http://ophid.utoronto.ca/mirDIP). Databases play a huge role in predicting target genes. miRanda (http://cbio.mskcc.org/miRNA2003/miranda.html), PicTar,^95^ miRbase (https://microrna.sanger.ac.uk/), TargetScan (http://www.targetscan.org/), miRDB (http://mirdb.org/), miRTarBase (http://miRTarBase.cuhk.edu.cn), miRWalk (https://www.umm.uni-heidelberg.de/apps/zmf/mirwalk/), MicroCosm Targets (https://www.ebi.ac.uk/enright-srv/microcosm), CircInteractome (http://circinteractome.nia.nih.gov/), TargetScanHuman (http://www.targetscan.org/vert_72/), Interactome (http://www.rna-society.org/raid/), and others can be used for target screening. A dual-luciferase assay was used to verify the target. Nygren et al.^96^ also reported a novel RenSP luciferase technique that, unlike firefly and Renilla luciferase, uses a target containing the GoClone structure as a light source. At present, the mainstream method to determine the relative expression level of miR-940 in cells, tissues, or body fluids by quantitative detection is qRT-PCR based on specific primers, that is, cDNA is synthesized first and then PCR amplification is performed. The qRT-PCR (stem-loop RT followed by TaqMan PCR analysis), invented by Chen and colleagues,^26^ using stem loop as primer, has the sensitivity, specificity and real-time detection ability of large dynamic range of PCR, and it has also been applied to detect the expression level of miR-940. In addition, nucleic acid molecular hybridization techniques such as *in situ* hybridization (ISH), fluorescence *in situ* hybridization (FISH), and northern blotting were also used to detect the expression level of miR-940, but only three experiments used it as a quantitative RNA detection method.^9^^,^^29^^,^^41^

## Conclusions

In this review, we found that miR-940 is dysregulated in more than 26 cancers. Among them, the miR-940 expression is upregulated in osteosarcoma,^11^ PAAD,^19^^,^^20^ and oral squamous cell carcinoma,^22^ including ESCC,^13^ and 22 other diseases.

In malignant tumors, including GC,^3^^,^^4^^,^^14^ HCC,^5^^,^^6^^,^^15^, ^16^, ^17^^,^^97^ NPC,^7^^,^^8^ and glioma,^9^^,^^10^ the altered pattern of miR-940 expression is controversial, which may be due to differences and limitations in the source of cell and tissue samples.

In addition, miR-940 is widely involved in cell proliferation, migration, invasion, apoptosis, EMT, cell cycle, osteogenic differentiation, and other biological processes by downregulating the expression level of target genes. By summarizing the molecular functions of target genes, we provide ideas for the study of the mechanism by which miR-940 influences these biological processes. The five signal transduction pathways by which miR-940 acts include the Wnt/β-catenin pathway,^11^^,^^19^^,^^27^ MAPK pathway,^46^ PD-1 pathway,^3^ and the PI3K-Akt pathway.^24^^,^^47^

Finally, there are many research results that have proven that miR-940 has great diagnostic potential and prognostic value, and it also has an impact on the efficacy of the drug.

However, current research still has some deficiencies. First, the sample size and source range should be expanded to accurately determine the expression level of miR-940 in each disease or disease stage. Second, the mechanism by which miR-940 influences cell biological processes remains to be studied. In addition, the role of miR-940 in diagnosis and prognosis needs to be further explored. There is a lack of experimental evidence to verify the value of miR-940 in diagnosis and prognosis. Finally, more experiments at the cellular and subcellular levels are needed to explore prove its auxiliary role in disease treatment.

The current research results have proven that miR-940 has a high value in the diagnosis and prognosis of diseases. In the future, it is necessary to further explore the tumor mechanism and pathways of miR-940, find the relationship between it and target genes, and verify its interaction with therapeutic drugs.

The value of miR-940 in cancer diagnosis and prognosis has been preliminarily discovered. In future studies, by refining the types and subtypes of cancer, miR-940 will have the opportunity to become a clinical tool for cancer diagnosis and prognosis judgment in the future. Recent studies on miR-940 and drug resistance in cancer provide a basis for future treatment strategies. miR-940 has the potential to make a significant contribution in the fight against cancer.

## Search strategy and criteria

The literature selection was performed in PubMed. The search item we used for relevant articles was “miR940 or miR-940 or microRNA940 or microRNA-940.” Inclusion criteria in our review were (1) research on cancers of humans, (2) research articles, and (3) articles published from 2014 to August 2020.
